# Remote assessment of cognitive dysfunction in hematologic malignancies using web‐based neuropsychological testing

**DOI:** 10.1002/cam4.5331

**Published:** 2022-10-11

**Authors:** Oscar Y. Franco‐Rocha, Misty L. Mahaffey, William Matsui, Shelli R. Kesler

**Affiliations:** ^1^ Brain Health Neuroscience Lab School of Nursing, University of Texas at Austin Austin Texas USA; ^2^ Department of Hematology/Oncology Stanford Cancer Institute, Stanford Health Care Palo Alto California USA; ^3^ Department of Oncology Dell School of Medicine, University of Texas at Austin Austin Texas USA

**Keywords:** cognitive function, hematological cancer, hematopoietic stem cell transplantation, psychosocial functioning, remote assessment

## Abstract

**Background:**

Cognitive impairment is a frequent adverse effect of cancer and its therapies. As neuropsychological assessment is not often standard of care for patients with non‐CNS disease, efficient, practical assessment tools are required to track cognition across the disease course. We examined cognitive functioning using a web‐based cognitive testing battery to determine if it could detect differences between patients with cancer and controls.

**Methods:**

We enrolled 22 patients with multiple myeloma (MM) or non‐Hodgkin lymphoma (NHL) and 40 healthy controls (mean age = 56 ± 11 years, 52% male). Participants completed the BrainCheck cognitive testing battery and online versions of select measures from the Patient Reported Outcome Measures Information System (PROMIS) during a video conference. MANOVA was used to compare BrainCheck and PROMIS scores between groups controlling for age and sex. An exploratory linear regression analysis was conducted within the cancer group to determine potential contributors to cognitive functioning.

**Results:**

All participants except for one control completed the online assessment measures without difficulty. Compared to controls, the cancer group demonstrated significantly lower scores in objective and subjective cognitive function, physical functioning, and social role performance and elevated fatigue scores. Corticosteroid treatment, immunotherapy, lower physical functioning, lower income, and older age significantly contributed to lower cognitive function (adjusted *R* ^2^ = 0.925, *F* = 19.63, *p* = 0.002).

**Conclusion:**

Remote assessment of cognitive and psychosocial functioning is feasible with patients with cancer following treatments. The BrainCheck cognitive testing battery has the potential to detect differences in cognition between patients with cancer and controls.

## Graphical abstract

Cancers and their treatments can result in impaired brain function. Evaluating brain function can be challenging due to feasibility and resource limitations. We remotely administered a web‐based, commercially available cognitive testing battery and demonstrated that patients scored significantly lower than controls. Immunotherapy, high dose steroids, older age, lower physical function, and lower income level were associated with lower cognitive function. Remote assessment of cognitive function is feasible in patients with cancer and could increase availability of brain function surveillance.

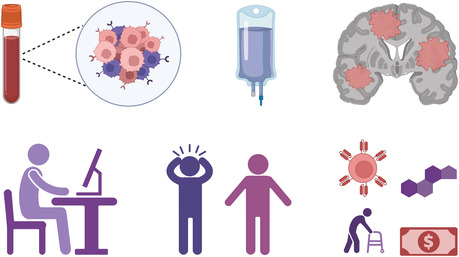

## 1 INTRODUCTION

Cancer and its treatments are frequently associated with decline in cognitive functioning. However, most studies to date have involved patients with breast cancer and therefore less is known regarding cognitive effects in other malignancies. Certain hematologic cancers, including multiple myeloma (MM) and non‐Hodgkin's lymphoma (NHL) undergo more intensive treatment regimens that may be associated with higher risk for cognitive decline.^1^ These treatments can include high dose chemotherapy, immunotherapy, high dose steroid, and hematopoietic stem cell transplant (HSCT) that may affect memory, attention, and executive function^2^, ^3^, ^4^ months and years after treatment completion,^5^ with worse cognitive outcomes when these treatment modalities are combined.^1^, ^6^

Cognitive functioning encompasses multiple abilities that are essential for treatment compliance.^7^ This is particularly relevant in hematologic malignancies as patients often remain on therapy for a long time, and cognitive impairment may reduce their ability to engage in treatment adherence.^8^ Cognition has also been associated with worse cancer outcomes. For instance, cognitive problems are associated with less self‐confidence, and more difficulties returning to work and with social relationships, which has the potential to reduce the quality of life of patients with cancer.^9^, ^10^ Furthermore, emerging studies have shown an association between cognitive impairment and lower survival in hematological cancers.^11^, ^12^

The incidence of hematological cancers varies depending on the type of cancer. For instance, NHL is one of the most common cancers in the United States, with a rate 1 in 42 for women and 1 in 52 for men.^13^ In contrast, MM is relatively uncommon, affecting 1 in 132.^14^ Thus, multi‐site or national recruitment may be needed to obtain adequate sample sizes to assess neuropsychological symptoms. In addition, cancer‐related cognitive impairment can be subtle in some patients, representing a decline from a previous level of functioning that may not be apparent with static testing. So, in future studies, longitudinal, repeated assessment is recommended to identify changes in neuropsychological symptoms over time.^15^ This can be challenging given the high cost of formal neuropsychological assessments and limited availability of neuropsychological resources within oncology centers. Remote measurement technology could provide a means of assessing cognitive functioning in a rapid and practical way. Nonetheless, to date, there have been few if any studies involving remotely administered cognitive testing batteries in patients with cancer. In this analysis, we aimed to (1) examine the feasibility of remotely assessing cognitive functioning in patients with hematologic cancer, and (2) determine if the testing battery could detect differences in cognition between patients with cancer and healthy controls.

## 2 METHODS

Feasibility studies help to assess whether methods and interventions are appropriate for further testing.^16^ Bowen et al. (2009)^16^ proposed general areas of focus in feasibility studies. The present feasibility study focused on implementation, the extent to which the testing battery was administered as planned. This included how often participants could access the online assessments without difficulty and how often they completed the assessments correctly.

### 2.1 Participants

We enrolled patients with MM or NHL who had received high dose chemotherapy and autologous HSCT at least 30 days prior. Patients were referred to the study by clinicians at the Livestrong Cancer Institute in Austin, Texas, and the Stanford Cancer Institute in Palo Alto, California from October 1, 2020, to November 1, 2021. We focused on hematologic malignancies that our study team had access to and that tend to receive intensive therapies while limiting the diagnoses to reduce sample heterogeneity. Patients were excluded for history of total body or cranial radiation. Medical history was obtained via patient self‐report. We also enrolled non‐cancer controls through referrals from enrolled patients, study clinicians, and social media postings. Participants were included if they were age 21 years or older, able to read, speak and write English, and had computer and internet access. Participants were excluded for prior history of diagnosed conditions known to affect cognition. The University of Texas at Austin Institutional Review Board approved the study (protocol# 2020‐05‐0117).

### 2.2 Cognitive assessment

Participants completed BrainCheck, a standardized, web‐based cognitive testing battery that assess processing speed, visual attention (Trails A), processing speed, visual attention and cognitive flexibility (Trails B), processing speed (Digit Symbol), response inhibition (Stroop), and verbal declarative memory (Immediate and Delayed Recall). These cognitive domains are of particular interest because, as previously mentioned, they are most frequently affected by cancer and its therapies, including patients with hematologic cancer.^2^, ^3^, ^4^ BrainCheck requires approximately 15 minutes to complete and has been shown to have strong psychometric properties and significant sensitivity for detecting mild cognitive impairment.^17^, ^18^ We previously demonstrated that this battery could detect mild cognitive impairment in COVID‐19 survivors^19^, ^20^ but it has not yet been used to evaluate cancer‐related cognitive impairment. BrainCheck provides standard scores for each test as well as for a Composite score (mean = 100, SD = 15), that are standardized for age and type of device based on normative data. To obtain the Composite score, a raw composite score is calculated first by averaging all assessment scores. Then, the raw composite score is normalized for age and type of device with normative data from BrainCheck. All scores are normally distributed (mean = 0, SD = 1) and then scaled to 100, with a standard deviation of ±15, with higher scores representing higher cognitive performance.^17^, ^18^

### 2.3 Patient reported outcomes

We also administered the Patient Reported Outcome Measures Information System v2.0 Cognitive Function Short Form 8a (PROMIS Cognitive) to measure subjective cognitive function.^21^ The PROMIS 57^22^ was administered to evaluate symptoms of depression, fatigue, anxiety, sleep disturbance, pain interference, physical functioning, and social role performance, which can contribute to cognitive effects. PROMIS measures were administered online via REDCap Survey (Vanderbilt, TN)^23^ using the REDCap Shared Library,^24^ which also automatically provided normative scores for each scale (mean = 50, SD = 10).

### 2.4 Assessment administration

After completing the screening process, the participants were emailed an invitation to an encrypted video conference call. During the video call, the research staff explained the study procedures and sent the link to the REDCap surveys. BrainCheck generates a link and anonymous login ID for each administration. Once the surveys were completed, the staff sent the BrainCheck link and ID to the participant so they could access the BrainCheck test. REDCap surveys include written instructions for completing each survey. BrainCheck includes written instructions for each test as well as practice versions to orient participants to the test procedures. Although BrainCheck and PROMIS are designed to be administered to examinees in a self‐directed manner, research staff remained on the video conference call, with screen sharing, to assist with any questions or technical issues and to help ensure an optimal and reliable testing environment. All participants completed the assessments at home. We required participants to use a laptop or desktop computer and the Google Chrome internet browser to reduce potential variation in testing platform.

### 2.5 Statistical analysis

Data were first examined visually for normality. Sample characteristics were compared between groups using t‐test and chi square tests, as appropriate. There was collinearity within BrainCheck and PROMIS scales, so MANCOVA was applied for each, controlling for age and sex. BrainCheck Composite score was evaluated separately using ANCOVA, also controlling for age and sex. We conducted an exploratory linear regression analysis within the cancer group to examine the effects of demographic, clinical, and psychosocial variables on cognitive function. Specifically, we included racial/ethnic minority status (minority = 1, non‐minority = 0), age (years), education (years), and sex (male = 1, female = 0) as these are known to contribute to cognitive performance in patients with cancer and other neurologic conditions,^25^, ^26^ corticosteroid treatment (1 = yes, 0 = no), immunotherapy (1 = yes, 0 = no), and post‐transplant days given prior studies demonstrating that these can affect cognitive function,^1^, ^3^, ^27^ and physical functioning score, fatigue score, and income level (1 = greater than $100 K, 0 = lower than $100 K) as these differed between groups. Cancer diagnosis (lymphoma = 1, myeloma = 0) was also included given that these have different pathologies and treatment regimens which may result in different cognitive outcomes. We examined only the BrainCheck Composite score to reduce multiple comparisons in our small sample. Alpha was set at 0.05. All analyses were performed using JASP v0.16.3 (JASP Team) or the R Statistical Package v4.2.0 (R Foundation).

## 3 RESULTS

### 3.1 Sample characteristics

During the year of our study, we enrolled 23 patients with MM (N = 11) or NHL. Eight of the patients with NHL reported a diagnosis of diffuse large B‐cell lymphoma while the others did not specify the type of NHL. Patients underwent autologous transplant 94.14 (SD = 62.48, range = 30–237) days, on average, prior to evaluation. All confirmed having received high dose chemotherapy, though only two specified which drugs, 36% reported receiving high dose corticosteroid (yes/no), and 23% reported receiving immunotherapy (yes/no). We also enrolled 40 controls. There were no significant differences between the groups in age, education, or biological sex (Table 1). In the cancer group, 32% endorsed racial/ethnicity minority status compared to 18% of controls, though this difference was not‐significant (X^2^ = 1.66, *p* = 0.197). All participants reported racial/ethnic status. Significantly more patients with cancer reported having an annual household income over $100 K compared to controls (X^2^ = 5.78, *p* = 0.016). However, six patients with cancer and four controls declined to report income information. Additionally, one participant attempted to enroll again under another name to obtain a second $25 e‐gift card honorarium, claiming their video camera was not functioning. Study staff recognized the individual's voice and the phone number so were able to prevent the fraudulent entry and this participant was administratively withdrawn from the study resulting in a final sample size of 22 in the cancer group.

**TABLE 1:** Sample Characteristics

	Cancer (*N* = 22)	Controls (*N* = 40)	Statistics	*p* Value
Age (years)	59.19 (11.87)	54.59 (9.53)	*t* = 1.67	0.101
Education (years)	16.09 (16.65)	16.65 (2.28)	*t* = 0.928	0.357
Male^a^	50%	52%	*X* ^2^ = 0.036	0.851
Racial/ethnic minority^b^	32%	18%	*X* ^2^ = 1.66	0.197
Income < $100 K	25%	61%	*X* ^2^ = 5.78	0.016
Multiple myeloma	50%			
Non‐Hodgkin's lymphoma	50%			
High dose dexamethasone	36%			
Immunotherapy	23%			

*Note*: Continuous data are shown as mean (standard deviation) and categorical data are shown as percentage.

^a^ Sex categories included male, female, non‐binary/third gender, prefer to self‐describe, prefer not to answer. All participants endorsed either male or female.

^b^ Racial/ethnic categories included Asian, Black, Caucasian, Hispanic/Latinx, Native American, Pacific Islander, prefer to self‐describe, prefer not to answer. No participants chose to self‐describe or not to answer. Racial/ethnic minority was defined as Asian, Black, Hispanic/Latinx, Native American, Pacific Islander, based on census data for the Palo Alto, CA and Austin, TX regions.

### 3.2 Feasibility

In terms of implementation, all participants were able to access the online questionnaires and cognitive testing without difficulty. All participants but one control completed the testing battery without difficulty. The control participant's session was interrupted by a phone call, resulting in the Trail Making Test timing out. Therefore, those data were excluded from analysis.

### 3.3 Cognitive performance

MANCOVA indicated a significant effect of group for BrainCheck tests (Pillai = 0.559, *p* < 0.001). All tests were significantly lower in the cancer group compared to controls, except for Trails B and Immediate Recall (Table 2). The ANCOVA for BrainCheck Composite score indicated significantly lower performance in the cancer group compared to controls (*F* = 29.16, *p* < 0.001).

**TABLE 2:** Cognitive performance controlling for age and sex

	Cancer Mean	%ile	Standard deviation	Control Mean	%ile	Standard deviation	*F*	*p*
Trails A	97.45	45	10.44	106.31	66	10.63	9.91	<0.001
Trails B	99.27	47	6.85	105.80	66	16.02	3.40	0.07
Digit symbol	87.59	21	15.31	108.51	73	12.56	29.43	<0.001
Stroop	89.82	25	13.50	106.86	68	12.90	13.37	<0.001
Immediate recall	106.41	66	11.70	110.25	75	5.48	3.08	0.08
Delayed recall	99.41	47	14.95	109.59	75	7.43	12.84	<0.001
Composite score	96.66	45	7.75	107.89	70	7.15	29.16	<0.001

*Note:* Scores have a normative mean of 100 and standard deviation of 15. Percentile (%ile) corresponds to the standard score and is interpreted as the percentage of individuals who score below the given standard score, on average, when those individuals are matched for age and sex.

### 3.4 Patient reported outcomes

MANCOVA indicated a significant effect of group for PROMIS scales (Pillai = 0.578, *p* < 0.001). Patients demonstrated significantly lower subjective cognitive function, physical functioning, and social role performance as well as significantly higher fatigue compared to controls (Table 3).

**TABLE 3:** Patient reported outcomes controlling for age and sex

	Cancer Mean	Standard deviation	Control Mean	Standard deviation	*F*	*p*
Cognitive function	46.65	6.78	52.00	7.80	7.79	0.01
Physical function	42.42	9.16	56.18	5.63	54.24	<0.001
Anxiety	51.15	6.05	50.07	7.96	0.33	0.57
Depression	49.75	5.49	47.13	9.21	1.43	0.24
Fatigue	52.96	6.62	44.82	7.08	24.15	<0.001
Sleep disruption	49.33	10.40	46.16	7.44	1.77	0.19
Social function	44.68	9.15	57.41	8.24	33.80	<0.001
Pain interference	52.56	10.27	48.51	6.93	3.26	0.08

### 3.5 Contributors to cognitive performance

The overall linear regression model was significant (adjusted *R* ^2^ = 0.925, *F* = 19.63, *p* = 0.002). Dexamethasone treatment (β = −28.58, *p* = 0.002), immunotherapy (β = −13.33, *p* = 0.009), lower physical functioning (β = 1.32, *p* = 0.002), lower income (β = 22.94, *p* = 0.005), and older age (β = −1.102, *p* = 0.022) were significantly associated with lower cognitive function.

Regression diagnostics indicated no violations of linearity, normality, or homoscedasticity. However, the high *R* ^2^ value in such a small sample suggested potential overfitting of the model. We then performed a 3‐fold cross‐validation of the regression model and observed that the mean *R* ^2^ value across the folds was much lower, *R* ^2^ = 0.551 (SD = 0.361), but explained over half the variance in Composite score. The *R* ^2^ value range across the folds explained 43%–76% of the variance.

## 4 DISCUSSION

We showed that remote cognitive assessment of patients with hematological cancers is feasible and provides several advantages including efficiency, convenience, and automated scoring. This is consistent with previous studies supporting the feasibility of remotely measuring cognitive performance in other populations.^19^, ^28^ Additionally, although for this pilot study we restricted enrollment to English‐speaking participants, BrainCheck can be administered in multiple languages. However, there are also several caveats to remote assessment. We supervised the testing sessions via videoconferencing and screen sharing, but it did not completely prevent interruptions in the participants' home environment. This occurred in only one case but happened even after we had instructed participants to complete testing in a quiet area, free from distractions. When using remote assessment, investigators must consider that some data might be lost due to uncontrolled factors in the participant environment, which would be much less likely in the typical laboratory or clinic in‐person scenario. Investigators must also be cautious with respect to recruitment considering our experience with the participant who attempted to enroll twice. Fraudulent enrollment may be a risk with remote assessment and therefore video conferencing is critical. However, this has certain technological requirements that may prevent subgroups of patients from participating.

Our findings also showed that the BrainCheck battery has the potential to detect differences in cognition between patients with cancer and healthy controls. The cancer group had significantly lower scores in executive function, attention, processing speed, and delayed verbal memory, which is consistent with other studies.^2^, ^5^, ^29^ Despite this, overall performance in the cancer group was clinically “average” for all tests except for Digit Symbol (processing speed) and Stroop (executive function), which were “low average,” higher than only 21%–25% of similarly aged individuals. This is consistent with previous studies suggesting that cancer‐related cognitive impairment tends to be mild to moderate.^30^

The cancer group performed significantly lower than the control group on all tests administered except for Trails B. Patients with cancer scored lower on this test than controls and thus, we may have lacked sufficient power to detect a difference here. Alternatively, this may have reflected a practice effect. Trails A is administered prior to Trails B and the two tests are highly similar. Although Trails B is supposedly more difficult than Trails A due to the addition of set shifting for measuring cognitive flexibility, Trails A may allow the examinee to become familiarized with the novel task and subsequently perform better on Trails B.^31^

Our exploratory regression analysis showed that several factors may contribute to cognitive impairment in patients with MM or NHL. Older age was associated with lower cognitive performance, consistent with previous findings.^26^ We also observed that lower income was a predictor of lower cognitive function. Income may be considered an indirect measure of cognitive reserve because it reflects the lifetime experience of individuals and their socioeconomic capacity^32^ including access to health insurance and higher‐quality medical care as well as stimulating environments, activities, and opportunities. On the other hand, education and racial/ethnic minority status were not significant contributors to cognitive function in our model, which contrast with previous findings.^33^, ^34^ This may suggest that income level is a more important predictor. However, our sample was small with most participants identifying as White and being highly educated and therefore, we may have lacked adequate power and variance to examine these effects.

Previous studies have proposed that corticosteroids and immunotherapy are risk factors for cognitive impairment in patients with hematologic cancers.^35^, ^36^ Even though the results from our study support this association, they must be interpreted cautiously. Again, our sample was small, only 23% of our participants received immunotherapy, and 36% received high‐dose corticosteroids. Future studies with larger and more diverse samples are necessary to analyze how clinical characteristics contribute to cognitive symptoms. Also, to explore differences in cognitive performance within different treatment types, such as the type of immunotherapy received.

Lower physical function was another predictor of cognitive performance and the only patient‐reported outcome associated with cognition in the present study. Physical function in this context refers to the individual's ability to carry out simple and complex physical activities, usually in social contexts.^37^ It is an important factor for promoting cognitive functioning and preventing cognitive decline.^38^ Furthermore, HSCT and concomitant treatment, like steroids, negatively impact physical functioning post‐transplant,^39^ which is consistent with our findings.

However, our results contrast with previous studies that showed an association between fatigue and cognitive performance in patients with breast cancer.^40^, ^41^ Post hoc analysis of our data indicated that women in the cancer group endorsed significantly more fatigue than men (*F* = 34.45, *p* < 0.001). However, given that there was no effect of sex on cognitive performance, the women may have been more resilient than the men with respect to brain health. Alternatively, we may have lacked adequate power for detecting an effect of fatigue on cognition.

Unlike prior studies, we did not detect an effect of education level on cognitive outcome.^26^, ^42^ Most previous studies have involved female patients with breast cancer, so they are not directly comparable to our findings. However, education level is considered a proxy for cognitive reserve and is thus frequently correlated with cognitive performance across conditions of brain health and disease.^32^ Our sample did not likely have adequate variance in education level to fully explore this relationship given that participants were all highly educated, with more than half the sample having college degrees.

Finally, we did not observe an effect of racial/ethnic minority status on cognitive function in this sample. Previous studies have shown mixed results on the influence of race and ethnicity in cognitive performance after cancer treatment. While some studies have found that racial/ethnic minority status is associated with cognitive performance,^25^, ^43^, ^44^ others have not.^45^, ^46^ However, the inclusion of racial and ethnic diverse groups in studies analyzing the influence of cancer and its treatment in cognitive health has been limited to date. Research studies analyzing these differences and the potential contributors of unique factors experienced by these groups, such as structural racism, are warranted.

This study was strengthened by restricting inclusion to participants with MM or NHL, which reduced the heterogeneity of the sample and shed light on the specific cognitive and psychosocial needs of patients with these types of cancers. However, the sample size was small, the participants were highly educated, and from upper class income, limiting the generalizability to patients with different sociodemographic characteristics. Another limitation was the significant number of variables assessed as potential predictors of cognitive functioning. There are always multiple potential contributors to the complexity of cognition with cancer treatments adding further to this. Reliance on patient‐report for treatment information resulted in missing data that may have provided further insights regarding contributors to cognitive function. Additionally, future studies with larger samples should include more precise questions regarding treatments such as the specific corticosteroid and immunotherapies received. The cross‐validation of our linear regression model produced a range of *R* ^2^ values that suggested the model had strong performance but was unstable. Further validation of the potential predictors of cognitive function that we have identified here is required. There may be other testing batteries with remote capability that yield different results and remote assessment is not a substitution for comprehensive clinical evaluation. Remote assessment requires access to technology that may limit or bias the sample, especially among socially disadvantaged patients who are at highest risk for cognitive effects.

In conclusion, remote assessment of cognitive and psychosocial functioning in patients with cancer is feasible. The BrainCheck cognitive testing battery has the potential to detect differences in cognition between patients with cancer and healthy controls and therefore could be more widely used in this population. Sociodemographic (age and lower income), clinical (corticosteroids and immunotherapy), and physical factors (lower physical function) may contribute to cognitive decline in people with MM or NHL after treatment. However, research studies with larger and more diverse samples are necessary to assess contributors to cognitive functioning.

## AUTHOR CONTRIBUTIONS

**Oscar Y. Franco‐Rocha:** Conceptualization (equal); validation (equal); visualization (equal); writing – original draft (equal); writing – review and editing (equal). **Misty L. Mahaffey:** Methodology (equal); supervision (equal); validation (equal); visualization (equal); writing – review and editing (equal). **William Matsui:** Methodology (equal); supervision (equal); validation (equal); visualization (equal); writing – review and editing (equal). **Shelli R. Kesler:** Conceptualization (equal); formal analysis (equal); funding acquisition (equal); methodology (equal); resources (equal); supervision (equal); validation (equal); visualization (equal); writing – original draft (equal); writing – review and editing (equal).

## FUNDING INFORMATION

This research was supported by funding from the National Institutes of Health (1R01CA226080, 2R01CA172145).

## CONFLICTS OF INTEREST

The authors declare no potential conflicts of interest with respect to the research, authorship, and/or publication of this article.

## Data Availability

All data relevant to the study are included in the article.
